# The Role of TOX in the Development of Innate Lymphoid Cells

**DOI:** 10.1155/2015/243868

**Published:** 2015-10-18

**Authors:** Corey R. Seehus, Jonathan Kaye

**Affiliations:** ^1^Research Division of Immunology, Departments of Biomedical Sciences and Medicine, Samuel Oschin Comprehensive Cancer Institute, Cedars-Sinai Medical Center, Los Angeles, CA 90048, USA; ^2^Department of Medicine, David Geffen School of Medicine, University of California, Los Angeles, CA 90095, USA

## Abstract

TOX, an evolutionarily conserved member of the HMG-box family of proteins, is essential for the development of various cells of both the innate and adaptive immune system. TOX is required for the development of CD4^+^ T lineage cells in the thymus, including natural killer T and T regulatory cells, as well as development of natural killer cells and fetal lymphoid tissue inducer cells, the latter required for lymph node organogenesis. Recently, we have identified a broader role for TOX in the innate immune system, demonstrating that this nuclear protein is required for generation of bone marrow progenitors that have potential to give rise to all innate lymphoid cells. Innate lymphoid cells, classified according to transcription factor expression and cytokine secretion profiles, derive from common lymphoid progenitors in the bone marrow and require Notch signals for their development. We discuss here the role of TOX in specifying CLP toward an innate lymphoid cell fate and hypothesize a possible role for TOX in regulating Notch gene targets during innate lymphoid cell development.

## 1. Introduction

Only relatively recently has it been discovered that the innate immune system has a wide range of effector functions carried out by a multitude of innate lymphoid cell (ILC) subtypes. Although the immune response mediated by these cells lacks antigen specificity intrinsic to the adaptive arm of the immune system, ILC responses are rapid and can be initiated by broadly expressed endogenous alarm signals from infected or damaged tissue (reviewed in [1]). In the adult bone marrow (BM), ILCs develop from common lymphoid progenitors (CLPs) through a Notch- [2–5] and Id2-dependent process [6, 7]. Like CD4^+^T_H_ cell subsets, ILCs are now classified by transcription factor expression and cytokine secretion profiles (reviewed in [8]). However, there is diversity within each group and some plasticity as observed for T-cells [1]. Thus, T-bet-dependent group 1 ILCs (ILC1s) secrete Th1-associated cytokines and are involved in the control of intracellular infections [9]. ILC1s also include natural killer (NK) cells. Group 2 ILCs (ILC2s) depend on the transcriptional regulators ROR*α*, TCF-1, and Bcl11b [3, 10–12] and like Th2 cells require GATA3 for their development and maturation [13]. ILC2s are important for tissue repair following influenza infection [14] and protection against helminths [15] and can modulate T_H_2 responses [16] and regulate fat metabolism [17]. ROR*γ*t-dependent group 3 ILCs (ILC3s) are the most diverse subtype. Group 3 ILCs include fetal lymphoid tissue inducer cells (LTi) that are required for lymph node organogenesis [18] as well as adult CD4^+^ LTi-like cells [19]. Other ILC3s express the natural cytotoxicity receptor (NKp46^+^) and are dependent on ROR*γ*t, TCF-1, and GATA3 for their development [20]. In addition, ILC3s regulate adaptive immunity [21] and promote intestinal homeostasis [22–24].

Identification of ILC-specific precursor cells has led to new insights into the transcriptional regulators involved in early ILC lineage specification. Expression of TCF-1 (encoded by* Tcf7*) was shown to identify ILC-specified progenitor cells called “early innate lymphoid progenitors” (EILPs) that are able to generate all ILC lineages [25]. EILPs do not have the potential to develop into T- and B-cells and are the earliest known ILC-specified progenitors. Reporter mice were used to identify a cell population defined by high* Id2* expression and termed the common progenitor to all helper-like innate lymphoid cells (CHILP). CHILP can differentiate into all ILC lineages, including LTi, with the exception of conventional natural killer (cNK) cells [9]. PLZF, a transcriptional regulator required for natural killer T (NKT) cell function [26], identifies a subset of CHILP that can differentiate into all ILC lineages with the exception of LTi and cNK cells [27]. This suggests that upregulation of PLZF and loss of LTi cell fate potential may mark further specification of the ILC lineage. The basic leucine zipper transcription factor NFIL3 is required for the development of cNK as well as all ILC subtypes [28–31]. NFIL3-deficient animals lack Lin^−^ *α*_4_ *β*_7_^+^ progenitor population (termed *α*LP for *α*_4_ *β*_7_ expressing CLP) that includes a minor subset of CXCR6^+^ cells that can give rise to all ILC lineages, including cNK [30, 32]. The exact nature of the relationship between *α*LP, EILP, and CHILP populations remains to be determined.

TOX (thymocyte selection-associated HMG-box protein) is a transcriptional regulator that was first identified in double positive thymocytes activated under conditions that mimic TCR signaling [33] and whose importance has been expanded to the innate immune system [34, 35]. TOX is a member of the superfamily of HMG-box proteins and a founding member of a smaller subfamily of four related proteins [36]. TOX has a prominent role in the development of the adaptive immune system (reviewed in [37]) and is expressed during multiple stages of mammalian corticogenesis [38]. Knockdown of TOX2 in human CD34^+^ progenitor cells from umbilical cord blood results in cNK maturation defects [39].* Tox2* is also expressed in CHILP [35], although the role of this TOX family member in ILC development is currently unknown. In addition, potential roles for TOX2 in reproductive organogenesis [40] and cancer [41] have been reported. TOX3 is involved in the regulation of neuron [41, 42] and oligodendrocyte [43] cell survival and has multiple roles in breast cancer [44], while TOX4, a ubiquitously expressed family member, interacts with a complex that controls chromatin structure and cell cycle kinetics [45].

**Figure 1:**
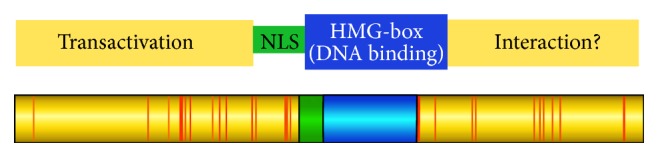
Domain structure of the TOX protein. Shown in red are differences between human and mouse TOX.

TOX family members contain an evolutionarily conserved DNA-binding HMG-box motif (reviewed in [37]) and, based upon amino acid sequence, are predicted to be members of the sequence-independent but structure-dependent HMG-box superfamily [36]. Recently, however, expression of a fusion of the TOX protein and bacterial DNA adenine methyltransferase in conjunction with deep sequencing (DamID) was used to identify potential TOX binding sites in the genome [38]. This approach led to identification of ~10,000 potential genomic TOX target sites, many associated with active enhancers. In addition, these data resulted in identification of a putative DNA-binding motif for TOX [38]. We have developed a binding assay for the DNA-binding domain of TOX, which reveals preferentially binding of this protein domain to distorted DNA when compared to linear DNA (J. Kaye, unpublished data). In addition, we have been unable to detect sequence-specific binding of the isolated HMG-box to the identified putative TOX binding motif. It is possible that TOX favors this motif only in the context of chromatin, that the full-length protein modifies the interaction with DNA, or alternatively that the motif is enriched in regions of chromatin with appropriate structure for TOX to bind.  Figure 1 shows domain structure of the TOX protein.

## 2. The Role of TOX in ILC Lineage Specification

**Figure 2:**
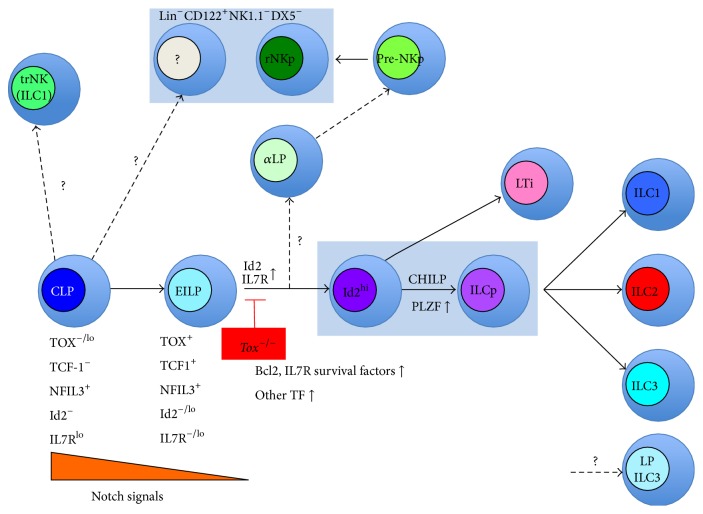
Proposed model of the role of TOX in innate lymphoid cell development. We propose that IL7R^lo^ cells represent a CLP to CHILP transitional cell population that requires TOX for progression. Notch signaling in CLP may initiate ILC differentiation but is terminated upon downregulation of Notch at the IL7R^lo^ transitional stage, and TOX could influence regulation of Notch gene targets. The NK cell lineage originates from a pre-CHILP stage (EILP), possibly via the *α*LP progenitor that is also TOX dependent. In the absence of TOX, rNKp cells fail to develop, but a population of Lin^−^CD122^+^ cells of unknown origin or function remains. Development of tissue resident NK cells is less affected by loss of TOX than are cNK cells, possibly indicative of a distinct pathway of development. Similarly, ILC3s are found in the gut of TOX-deficient mice, but whether this is due to a TOX-independent pathway of development or homeostatic proliferation is not clear.

All subtypes of ILCs can develop from CLP in the presence of Notch ligands [25] although the requirement for Notch signaling may differ between ILC group members. For example, ablation of RBPj*κ*, a key transcriptional regulator of Notch signaling, in adult mouse hematopoietic cells greatly reduced NKp46^+^ ILC3s but resulted in only a modest decrease in CD4^+^ LTi-like cell frequency in the gut lamina propria, while cNK cell numbers were unaffected [46]. For ILC2, retroviral transduction of the pan-Notch inhibitor dominant-negative Mastermind like-1 into adult multipotent BM progenitors resulted in a significant decrease of mature lung resident ILC2s, indicating an* in vivo* reliance on Notch for their development and/or survival [10]. Furthermore, in adult BM derived CLPs, Notch signals promoted ILC2 differentiation, although the presence of Notch ligands was only needed transiently to restrict differentiation to an ILC2 fate [3]. Moreover, CLPs isolated from fetal liver and adult BM were dependent on Notch for differentiation into an *α*_4_ *β*_7_^+^Ror*γ*t^−^ LTi-like cell precursor population, but Notch signals needed to be terminated for differentiation into mature ROR*γ*t expressing ILC3s and blocking a T-cell fate [2]. Unlike CLP, ILC-committed progenitors poorly express Notch proteins and mRNA [25, 32, 35] and thus a potential mechanism in limiting Notch signals during ILC lineage development. Indeed, development of ILC from EILP is Notch independent [25]. While the timing of Notch signals may be critical for differentiation of the ILC lineage and importantly may favor an ILC over a T-cell fate, the strength of Notch signaling may act as an independent regulator of cell fate [47].  Figure 2 shows proposed model of the role of TOX in innate lymphoid cell development.

In the absence of TOX, the lineage negative *α*_4_ *β*_7_^+^CD25^−^Flt3^−^ BM precursor population expressed low levels of the Notch target genes* Tcf7* [10],* Hes1* [48],* Gata3* [49], and* Bcl11b* [50], although surface Notch1 expression was normal in CLP [35]. Thus, it is possible that TOX is necessary for the downstream activation of genes that are direct targets of Notch signaling. In agreement with this, TOX was found to bind to regions of* Hes1* in human embryonic kidney cells [38].* Hes1* is important for early T-cell specification in the thymus as conditional deletion of* Hes1* in hematopoietic progenitors results in lower thymic cellularity and a higher proportion of immature B-cells in the DN population [48]. Interestingly, we have observed a predisposition of TOX-deficient CLPs to differentiate into CD19^+^B220^+^ B-cells on OP9-DL1 stromal cells in a number of experiments, although the effect was highly variable precluding statistical verification (J. Kaye, unpublished results).* Hes1* deletion in fetal liver progenitor cells does not impact ILC2 development [10] suggesting other essential factors are downstream of Notch signals, likely TCF-1.

Details of the CLP to CHILP transition remain poorly defined. Using reporter strains of mice, expression of TOX and Id2 is near coincident in the lineage negative CD25^−^ subset of *α*_4_ *β*_7_^+^IL7R^+^ BM progenitors that includes CHILP, and these cells were reduced in* Tox* ^−/−^ animals [35]. Whole transcriptome sequencing of the remaining precursor population in TOX-deficient mice revealed lack of expression of key transcriptional regulators implicated in ILC development as well as a significant decrease in* Id2* and* Il7r* expression [35]. We have suggested that IL7R^lo^ cells may be in transition from CLP to CHILP, a cell population that fails to progress in the absence of TOX. Indeed, we have preliminary evidence that such cells may exist in wild-type mice and that TOX may precede even Id2 expression (J. Kaye, unpublished data).

As indicated above, where *α*LP cells fit into the CLP to CHILP transition is not clear. In adult BM progenitors,* Tox* is upregulated in *α*_4_ *β*_7_^+^CXCR6^−^ cells and is concomitant with* Nfil3* expression [32]. The CXCR6^+^ population of *α*LP is missing in the absence of TOX (J. Kaye, unpublished data) and* Cxcr6* was poorly expressed in a TOX-deficient ILC precursor population while* Nfil3* expression was not affected [35]. NFIL3 has been shown to directly bind to the TOX promoter in a mouse lymphoma cell line and ectopic expression of TOX restored ILCs in the absence of NFIL3, though with low efficiency [30]. NFIL3 is expressed in CLP and without this factor, the very low levels of* Tox* present in CLP were reduced. Whether NFIL3 is necessary for TOX upregulation during the CLP to CHILP transition, however, remains to be determined.

Regulation of TOX may not be the only function of NFIL3. Indeed, NFIL3 was shown to directly bind to the* Id2* gene in CHILP and enforced* Id2* expression in NFIL3-deficient fetal liver *α*_4_ *β*_7_^+^ precursors rescued ILC and NK development [31]. Interestingly, TOX reconstitution was not sufficient to rescue NFIL3 deficiency in CD4^+^ LTi-like cells [31], suggesting a more complex relationship of these transcriptional regulators that may depend in part on the progenitor population being studied. It is also surprising that, in the absence of NFIL3, *α*LP and CHILP are severely reduced but lymph nodes are still present [31], while TOX-deficient or Id2-deficient mice lose both CHILP and lymph nodes [6, 34]. The failure of NFIL3 deficiency to phenocopy either Id2 or TOX deficiency may reflect differences in the regulation of Id2 and TOX in the fetus and adult. Alternatively, NFIL3 may be one of a number of factors to regulate TOX and Id2. In the absence of NFIL3, a modest reduction in both Id2 and TOX could be sufficient to prevent efficient CHILP formation but still allow sufficient LTi cell development to promote lymph node formation. Clearly, the exact relationship between TOX, Id2, and NFIL3 during early ILC development must await additional experimentation, but it is clear that expression of these early factors, along with TCF-1 (encoded by* Tcf7*) and* GATA3*, is key mediators of ILC lineage specification.

How signaling pathways may regulate the expression of TOX during ILC development in the bone marrow is not known. We found that TOX is induced by TCR-mediated calcineurin signaling during positive selection in the thymus [51]. Interestingly,* Tox* expression in the brain is also regulated by calcineurin via activation of NFAT4 [38]. Whether calcineurin also plays a role in TOX regulation during ILC development, however, remains to be determined.

It is possible that TOX is required for the regulation of prosurvival factors early in ILC development, including IL7R and Bcl2 [35]. With the exception of most cNK, all ILCs express IL7R.* Il7* deficient mice have normal numbers of CLP and CHILP-like precursors but ILC2 specific progenitors are compromised [9]. In addition, intestinal NKp46^+^Ror*γ*t^+^ ILC3s are severely reduced in the absence of* Il7*, yet cNK cells remain unaffected [52]. These data suggest important roles for IL-7 signaling during ILC development. Isolated* Tox* ^−/−^ ILC precursor cells express less surface IL7R*α* and* Il7* message than CHILP [35]. Moreover, despite normal IL7R*α* surface expression on CLPs, TOX deficiency results in defects in cell survival and/or proliferation when CLPs were differentiated into ILC* in vitro* [35]. TCF-1, a transcription factor whose expression is required for the development of multiple ILC subtypes [20, 53], is poorly expressed in TOX-deficient ILC precursors [35]. Furthermore, in mature ILC2s, TCF-1 regulates the* Il7r* gene [10], possibly one mechanism by which TOX could indirectly regulate IL7R expression.

## 3. Complexity in the Role of TOX in ILC3s

ILC3s are likely the most diverse ILC population, with additional complexity due to plasticity of these cells [54].* Tox* is expressed in LTi cells [34] and in a heterogeneous manner in other ILC3 populations by reporter [35]. Fetal liver LTi cells [34], splenic ILC3s, NK1.1^+^NKp46^+^ ILC3s, and adult CCR6^+^ LTi-like ILC3 populations are reduced in the absence of TOX [35]. The small intestine lamina propria (LP) contain CCR6^+^ fetal-derived LTi-like cells and a heterogeneous mix of CCR6^−^ postnatal ILCs [55]. Interestingly, the frequencies of the major populations of small intestine LP ILC3 are normal in adult* Tox* ^−/−^ mice and cell recoveries indicated the possibility of cell expansion of some ILC3s in the absence of TOX [35]. These data may suggest a TOX-independent pathway of gut-resident ILC3 development, including reliance on distinct progenitor cells. LP contains CLP-like cells that can give rise to NK and ROR*γ*t^+^ cells in culture [32]. In addition, non-LTi, arginase-1^+^ ILC precursors that express Id2 as well as GATA-3, T-bet, and ROR*γ*t have been identified in the fetal gut and are absent in adult BM [56]. These cells can differentiate into ILC1s, ILC2s, and ILC3s* in vitro* but their dependence on TOX is unknown. Interestingly, the loss of transcriptional regulators necessary for ILC development has less of an effect on the development of ILC3 than other ILCs. For example, PLZF^hi^ progenitor cells can produce ILC3* in vivo*, but the transcription factor itself is required for ILC2 and some ILC1 but not ILC3 development [27]. Also, TCF-1 deficiency results in a reduction of NKp46^+^ ILC3 but not other ILC3 subsets, suggesting potential heterogeneity in the molecular regulation of distinct ILC3 subtypes [20]. NFIL3 is not required for lymph node organogenesis [28], and the loss of ILC3 in the absence of NFIL3 is variable across independent reports [28–30]. Therefore, the relative contribution of adult ILC BM progenitors to ILC3 development in the LP is not clear, but ILC progenitor populations that do not require TOX for their development may exist.

Alternatively, it is also possible that in the gut microenvironment a small population of ILC3s that do develop can expand in the absence of TOX. It has previously been reported that a proportion of CCR6^−^T-bet^+^ ILC3s in LP are highly proliferative [55]. Furthermore, ILCs present in the small intestine LP have a transcriptome that is correlated with activated cells when compared to ILCs in other locations [57]. It is also possible that expansion of LP ILC3s may be pronounced in* Tox* ^−/−^ mice, due to a block in CD4 T-cell generation, which includes a block in Treg development [58]. Whether or not adult ILC3 subtypes are derived from CLPs that migrate to the small intestine LP and expand into ILC3 lineages in the absence of TOX remains to be determined.

## 4. The Conundrum of NK Cell Development

NK cells develop from BM resident CLPs and pass through a series of stages defined by cell surface molecules and functional activity. NK cell development is dependent on the transcriptional regulators NFIL3, Id2, and TOX. Previously, we showed that TOX was highly expressed in immature NK cells (iNK) and mature NK (mNK) and expression coincided with that of Id2 [34, 35]. In the absence of TOX, NK development is blocked subsequent to the NKp stage, consistent with loss of NK-specific killing* in vivo* [34]. Like TOX, Id2 has been shown to be required for the development of cNK cells but does not affect the NKp stage of development [59]. Similarly, NFIL3-deficient mice were shown to have reduced numbers of iNK and mNK cells but normal NKp numbers [60]. In addition, Nfil3 was shown to bind directly to Id2 and Eomes regulatory regions and ectopic expression of Eomes, T-bet, and Id2 rescued Nfil3 deficiency to varying levels although TOX did not [61].

The late block in cNK development in the absence of TOX, Id2, and NFIL3 is difficult to reconcile. Recent results show an early requirement for these factors in multipotential ILC progenitor development, including *α*LP in the case of NFIL3 and TOX. However, this issue has been resolved at least in part by a more refined definition of NKp cells (rNKp), which represents a minor subpopulation of previously defined NKp cells, as well as identification of an earlier pre-NKp stage [62]. Thus, NFIL3 deficiency results in reduced numbers of pre-NKp cells [61]. In the absence of TOX, rNKP cells are lost although the effect on pre-NKp cells remains to be determined (J. Kaye, unpublished Data). However, a significant population of CD122^+^NK1.1^−^DX5^−^ cells remains, consistent with earlier findings [34]. The lineage identity of these cells or their precursor potential remains unknown.

In addition to cNK cells, a noncirculating CD49a^+^ tissue resident population of NK cells (trNK) has been identified in multiple tissues [63]. Interestingly, trNK cells are not dependent on NFIL3 for their development and/or maintenance [63]. To date, a specific trNK cell progenitor has not been identified, but these cells share many similarities with ILC1 and likely come from an ILC progenitor population [64]. Interestingly, cNK cells are more affected by TOX deficiency than are trNK cells in the liver consistent with a distinct pathway of development (J. Kaye, unpublished Data).

## 5. Conclusions

The discovery of innate lymphoid cells has greatly expanded our knowledge of the immune system. TOX, a transcriptional regulator that had been previously associated with CD4 T-cell development in the thymus, is also a key regulator of innate lymphoid cell development. Specifically, TOX is required for differentiation of CLP to early ILC progenitors. In the absence of TOX, the remaining progenitor cells poorly express almost all known transcriptional regulators involved in development of the ILC lineage, suggesting that TOX may be among the earliest factors driving ILC lineage specification. Whether TOX may also play a role in mature ILCs is under investigation.

Data suggest that transient Notch signaling may play a role in ILC lineage specification. We have shown that CHILP poorly express all Notch genes, suggesting one mechanism by which Notch signaling could be extinguished. TOX-deficient progenitors fail to upregulate Notch target genes, but whether TOX can directly impact Notch-mediated gene regulation remains to be determined. In addition, we observed poor expansion and/or survival of TOX-deficient CLP when differentiated toward the ILC lineage in culture. This may suggest an additional role for TOX in progenitor cell survival, consistent with loss of* Bcl2* in TOX-deficient progenitors.

The small intestine lamina propria consists of a diverse ILC3 population, some of which are present in the absence of TOX. How these cells remain in the face of a severely reduced CHILP population is unclear. Similarly, many questions remain concerning the pathways of generation of cNK and trNK cells, as well as an unknown population of TOX-independent CD122^+^ cells, and where NK cell progenitors split from the helper-like ILC lineages. Thus, whether the established CLP to CHILP paradigm of bone marrow progenitor development can explain all ILC development must await further experimentation.
